# Implementing a school-based mental health literacy programme for adolescents: barriers, facilitators and preliminary outcomes

**DOI:** 10.1093/heapro/daaf236

**Published:** 2026-01-13

**Authors:** Savita Gunasekaran, Shazana Shahwan, Wei Jie Ong, Benedict Wei Zhi Lim, Hui Nee Jennifer Koh, Yoke Boon Tan, Yen Sin Koh, Meiyi Stella Teo, Chiang Chiang Anita Lim, Mythily Subramaniam

**Affiliations:** Research Division, Institute of Mental Health, 10 Buangkok View, Buangkok Green, Medical Park, Singapore 539747, Singapore, Singapore; Research Division, Institute of Mental Health, 10 Buangkok View, Buangkok Green, Medical Park, Singapore 539747, Singapore, Singapore; Research Division, Institute of Mental Health, 10 Buangkok View, Buangkok Green, Medical Park, Singapore 539747, Singapore, Singapore; Research Division, Institute of Mental Health, 10 Buangkok View, Buangkok Green, Medical Park, Singapore 539747, Singapore, Singapore; Impact & Research, Transformation Office, TOUCH Community Services, 3615 Jalan Bukit Merah, Gateway Theatre Level 5, Singapore 159461, Singapore, Singapore; Research Division, Institute of Mental Health, 10 Buangkok View, Buangkok Green, Medical Park, Singapore 539747, Singapore, Singapore; Research Division, Institute of Mental Health, 10 Buangkok View, Buangkok Green, Medical Park, Singapore 539747, Singapore, Singapore; Impact & Research, Transformation Office, TOUCH Community Services, 3615 Jalan Bukit Merah, Gateway Theatre Level 5, Singapore 159461, Singapore, Singapore; Impact & Research, Transformation Office, TOUCH Community Services, 3615 Jalan Bukit Merah, Gateway Theatre Level 5, Singapore 159461, Singapore, Singapore; Research Division, Institute of Mental Health, 10 Buangkok View, Buangkok Green, Medical Park, Singapore 539747, Singapore, Singapore; Saw Swee Hock School of Public Health, National University of Singapore, 12 Science Drive 2, Singapore 117549, Singapore, Singapore

## Abstract

A novel school-based mental health literacy (MHL) programme titled ‘Do You M.I.N.D.?’ was implemented in Singapore to improve knowledge, attitudes, and help-seeking among secondary school students. The present study examined its implementation barriers, facilitators, and preliminary impact on students’ MHL. A mixed-methods design was used. Qualitative data were collected through semi-structured interviews with 22 key informants and six session observations guided by the Consolidated Framework for Implementation Research. Quantitative data were collected through a 21-item pre- and post-intervention questionnaire with 841 Secondary One students. Pre-post differences were analysed using Wilcoxon signed-rank tests and difference-in-difference (DiD) analyses, with a significance level set at 0.05. Findings revealed that key facilitators included the relative advantage of the interactive sessions that incorporated Virtual Reality, understanding students’ mental health needs and resources, engaging school administrators, and executing with implementation fidelity and fit. However, this relative advantage was lost when the programme shifted to Zoom video conferencing during the COVID-19 pandemic, which served as a salient barrier alongside implementation complexity, insufficient available resources, and challenges in engaging students and teachers. Pre-post data showed overall improvements in MHL, with face-to-face delivery yielding significantly better scores for two items. The programme shows promise in enhancing students’ MHL, with the study providing insights for refining content and implementation strategies. The reduced effectiveness during online delivery underscores the importance of maintaining interactive elements in future adaptations. These results highlight the need for sustained resources, stakeholder engagement and support, and delivery models that preserve programme interactivity to optimize outcomes.

Contribution to Health Promotion

- Examined implementation factors and effectiveness of a school-based mental health literacy programme (MHL) for adolescent students in Singapore that incorporates virtual learning and interactive activities.
- Addresses the need for early mental health education in Asia, where stigma and low help-seeking remain prevalent among adolescents.
- The evaluation included pre-post student MHL outcomes, ethnographic data and perspectives from stakeholders delivering the programme, offering valuable insights.
- Contributes to the limited research on mental health education and promotion in Asian schools.
- Highlights practical ways to implement and supports the case for scalable tech-based MHL programmes.

## Background

There is growing awareness of the prevalence of mental health conditions among adolescents, with evidence showing that about half of all cases begin before the age of 14 (García-Carrión *et al*. 2019, Santre 2022). The World Health Organization (WHO) revealed that ∼14% of adolescents aged 10–19 experience mental health conditions; yet, these issues often go unnoticed and unaddressed (WHO 2024). Some studies investigating barriers to help-seeking among youths have identified negative beliefs and fear of others’ reactions as prominent factors (Rickwood *et al*. 2005, Rowe *et al*. 2014), while others have cited lack of knowledge about mental health and help-seeking avenues (Reardon *et al*. 2017, Radez *et al*. 2021).

Given the prevalence and onset of mental health conditions and barriers to seeking care, youths are an important target group for Mental Health Literacy (MHL) interventions (Nazari *et al*. 2024). Additionally, compared to adults, adolescents’ attitudes are pliable, providing an ideal opportunity to promote MHL (Corrigan and Watson 2007). MHL is defined as the ‘knowledge and beliefs about mental disorders which aid their recognition, management or prevention’ (Jorm *et al*. 1997). Core components of MHL include (i) the ability to identify specific mental disorders; (ii) understanding of associated risk factors and causes; (iii) knowledge of effective self-help strategies; (iv) awareness of available professional help; (v) attitudes that promote recognition of mental health issues and appropriate help-seeking; and (vi) knowledge of how to access reliable mental health information (Jorm 2000). Increasing youths’ MHL enhances their awareness about mental health problems, such as recognizing conditions, help-seeking efficacy, and the ability to dispel misinformation that perpetuates stigma (Jorm *et al*. 1997, 2007, WHO 2021).

Singapore is a Southeast Asian country with a population of 5.45 million as of June 2021 (Singapore Department of Statistics 2021). Analogous to findings from the aforementioned studies, an epidemiological study reported that 14.9% and 27.0% of residents aged 15–35 experienced severe to very severe symptoms of depression and anxiety, respectively (Subramaniam *et al*. 2025). Furthermore, a local study by Pang *et al*. (2017), using the Attitudes Towards Serious Mental Illness scale, found that youths aged 14–18 showed high stigma and low social tolerance, with about half reporting that they would feel embarrassed if diagnosed with a mental illness, and one-third believing that their friends would view them as weak. Additionally, only one in four could name specific mental illnesses (Pang *et al*. 2017).

In recent years, several initiatives have been launched to promote adolescent mental well-being in Singapore. For example, the Response, Early Intervention and Assessment in Community Mental Health programme partners with schools to support youths in managing mental health symptoms (Ministry of Health 2023). However, most initiatives focus on post-diagnosis intervention, with limited educational efforts targeting youths before issues emerge or escalate. Pilot school-based MHL programmes have been implemented locally but primarily target university-aged students (Goh *et al*. 2021 , Tay *et al*. 2022). To address this gap, a novel school-based MHL programme, ‘Do You M.I.N.D.?’ (DYM), was introduced to target secondary school students aged 13–17. It aims to educate youths about mental health through Virtual Reality (VR) scenarios and experiential activities. It combines technology-driven engagement with evidence-informed content, standing out as a pioneering digital MHL programme that addresses the needs of today’s digitally native youths.

Nazari *et al.* (2023) emphasized the need to assess MHL interventions to determine their effectiveness and enhance their cost efficiency. There is a growing need for implementation research to examine the feasibility and sustainability of MHL interventions aimed at adolescents (Marinucci *et al*. 2023). Given the novelty of the DYM programme, the present study examined key implementation barriers and facilitators to assess its feasibility and scalability, alongside preliminary outcome data to assess its effectiveness. Following similar work (e.g. Brooks *et al*. 2023, Sadler *et al*. 2024), a mixed-methods approach was used. The Consolidated Framework for Implementation Research (CFIR) (Damschroder *et al*. 2009) guided analysis of individual and organizational level factors, while pre-post questionnaires provided preliminary evidence of the programme’s potential to improve MHL. Quantitative results were treated as early indicators, with implementation outcomes and context as the primary study focus.

## Methods

### Intervention details

**‘**Do You M.I.N.D.?’ (DYM) is a youth mental wellness programme launched by TOUCH Community Services (referred to as ‘the organisation’ henceforth) in October 2017 for secondary school students in Singapore. The programme focuses on depression, anxiety, eating disorders, and self-harm, guided by evidence from the Singapore Mental Health Study and common youth counselling cases observed by the organisation. The programme aims to promote mental well-being and enhance youths’ awareness of mental health issues and perceptions towards people with mental health conditions. It employs experiential learning and VR to equip youths with knowledge about mental health, illustrate challenging situations that people with mental health conditions face, show them how they can support their peers, and help them gain a more accurate understanding of the recovery process.

Participants complete four learning stations tailored to each mental illness, along with a dedicated VR station for depression, in a round-robin style. At each station, participants engage in interactive and engaging activities, with mental health messages as part of the structured learning and reflection process.

The VR experience was developed by the organisation’s mental health programme team, comprising experienced mental health professionals, who conceptualized the content based on their experience with youths, insights from individuals with lived experience of depression, and evidence from the literature. The organization then collaborated with two local vendors to refine the storyboard, strengthen character interactions and dialogues, and ensure the intended learning outcomes. The vendors filmed the VR video using high-resolution equipment, with narration provided by a youth volunteer in recovery, and characters were played by the organisation’s staff and youth volunteers. The final video was reviewed by the programme team, management, the youth narrator, and the vendors, and refined based on key feedback. The VR experience employs a ‘choose your own adventure’ concept, where participants make choices for a fictitious character who suffers from depression. The choices have an impact on how the symptoms of depression play out for the character. The VR video was hosted on Storyhive, an online VR platform developed by one of the vendors. To access the video, each participant used a Samsung Gear VR headset powered by Oculus technology and connected to a compatible Samsung smartphone. The phone was logged onto Storyhive to display and play the video; the VR headset held the phone and enabled the users to participate in the experience via control buttons. Participants experienced scenarios such as the character’s daily struggles and interactions with community members.

Additionally, through a facilitated discussion and reflection segment, participants learned coping methods that can help them manage issues affecting them. They also picked up tips on how they may support their peers who may be struggling with mental health issues and learned about existing resources in the community. In total, each intervention session lasts for about 3 hours. A brief intervention outline is available in the Supplementary materials.

The intervention is designed to strengthen adolescent MHL by combining immersive VR technology with interactive, station-based activities that translate these concepts into relatable experiences. These activities are expected to improve recognition of common mental health issues, reduce stigma, and enhance awareness of positive coping strategies and confidence in noticing early warning signs. In turn, these short-term outcomes are anticipated to foster intermediate changes, including stronger peer support, earlier detection of symptoms, and increased openness to conversations and help-seeking. Ultimately, the long-term goal of the programme is to achieve sustained improvements in MHL, encourage earlier intervention, and contribute to a supportive school culture that normalizes mental health awareness and help-seeking.

According to the WHO (2021), youths at this developmental stage demonstrate stronger perspective-taking abilities and begin to integrate values, ethics, and ideologies into their behaviour. This created an opportunity for the intervention to engage participants in activities that promote empathy and acceptance by offering first-person perspectives. As adolescents’ cognitive abilities become more concrete, they can develop basic MHL, such as understanding the prevalence and causes of mental illnesses and distinguishing everyday mental health challenges from clinically diagnosed disorders (WHO 2021), which the intervention addresses.

Furthermore, the intervention is delivered using simple, clear language for youths, including relatable scenarios and local examples. Stations were run by trained mentors who were recruited using several strategies. Before facilitating sessions, mentors attended group training that involved reviewing the verbatim manual guide and discussing the mental health conditions to familiarize themselves with the content. The mentors underwent mock facilitation, followed by shadowing an experienced mentor for 2–3 sessions before they ran sessions on their own.

### Study details

The present study utilized a convergent parallel design mixed-methods approach, examining programme implementation by exploring stakeholder perspectives (qualitative) and measuring student outcomes (quantitative). The participating schools were identified by the organization based on schools that provided permission for research participation. Consent was obtained from school administrators to allow interviews with key school stakeholders, observation of programme delivery, and collection of pre- and post-intervention data from students. Individual informed consent from participants, as well as school-wide consent, was secured in line with ethical requirements. Ethical approval for the study was provided by the National Healthcare Group Domain Specific Review Board (IRB protocol number: 2019/01101).

### Qualitative data

Data were collected from semi-structured interviews (SSIs) and ethnographic observations. SSIs with stakeholder groups provide unique perspectives, particularly information about personal experiences, organization dynamics, the setting, and how its characteristics may have affected implementation. Varying stakeholder perspectives are also important to provide insights during different phases of the intervention. However, Green *et al*. (2015) highlighted that stakeholders may be limited by personal biases or subjectivity. As such, ethnographic data (i.e. observations) collected by researchers external to the delivering organization provided an objective lens and insights about environmental factors that stakeholders might not articulate (Green *et al*. 2015). Data collection and analysis were based on the five domains of the Consolidated Framework for Implementation Research (CFIR) (Damschroder *et al*. 2009). Refer to Table 1 for the breakdown of the number of interviews and observations.

**Table 1:** Number of (a) interviews per stakeholder group and (b) observations per site.

	Number (*n*)
(a) Interviews	
Mentors	10
School Liaisons	4^a^
Organization Staff	8
(b) Observations	
School 1	2
School 2	1
School 3	1
School 4 (Zoom)	1
School 5 (Zoom)	1

^a^During one of the School Liaison interviews, there was a technical issue with the audio recorder, resulting in the absence of the audio file and transcription. Interview notes which were taken during the interview were utilized.

#### Participants

Participants were from three stakeholder groups: staff/management from the organisation that were involved in the conceptualization and design of the programme (Organisation Staff), teachers or counsellors who were involved in the inception of the programme into schools (School Liaisons), and organisation staff and volunteers who facilitated the programme execution in schools (Mentors). Data collection ended after 22 SSIs as data saturation was reached (Braun and Clarke 2021).

#### Data collection

Interviews were conducted face-to-face and subsequently via Zoom video conferencing from March 2020 to November 2021, guided by an SSI guide. Interviews were audio-recorded and transcribed verbatim for analysis. Additionally, a total of four face-to-face observations and two Zoom observations of the programme were carried out at five secondary schools. Observations were conducted in pairs for face-to-face sessions and by four observers for Zoom sessions to minimize observer bias and ensure that documentation was as complete as possible. The observations were documented using a guide developed with reference to the programme’s verbatim manual. The guide comprised seven sections: five sections that contained station-specific prompts on what to observe, a section consisting of a checklist for the Q&A (Question and Answer) (e.g. did the mentor respond clearly), and a final section with general prompts (e.g. how adequate was the logistical arrangement). Observers completed a fidelity checklist and wrote field notes (descriptions of observations). After each observation, the observers reconvened and shared their immediate reflections and compared notes. The team discussed what was observed, including whether there were any patterns seen, and any incongruencies between observers were highlighted and resolved. The observations were analysed by comparing them with content identified in the SSIs.

#### Data analysis

Data was analysed using a deductive approach, partially guided by the consensual qualitative research method (Hill *et al*. 1997, Hill 2012). Interview guides tailored to each stakeholder group were developed before data collection. Using CFIR as a guide, study team members (MS (MBBS, PhD), SS (MClinPsych), WJO (BA), BWZL (BA), SG (BScHons), YBT (BSocSci)) developed potential questions by translating the constructs into open-ended, context-sensitive prompts. CFIR constructs that were not relevant to the research aims and did not fit into any overarching category were excluded. Team members drafted the questioning route, reordered, and paraphrased the questionnaire to generate a logical flow. Decisions to exclude questions were based on their relevance in eliciting responses to the research objective, and all final decisions were made by the lead investigator (MS). An excerpt of the interview guide is provided in the Supplementary Materials.

Team members independently analysed a subset of the SSI transcripts and observation notes and identified and generated key constructs from the CFIR. All members had formal training in implementation frameworks. During this preliminary analysis, study team members familiarized themselves with the data and highlighted quotes of relevance, which could be grouped into axial codes, identifying the constructs that were representative of the data. A preliminary codebook was generated with the shortlisted 22 CFIR constructs. Finally, consensus on any disagreements regarding codes and themes was reached through discussions and iterative review, and a codebook was thereby developed. The codes were specified with the following: label, definition, inclusions and exclusions, and typical and atypical exemplars from raw data. The codebook was refined, and constructs from the CFIR that did not have many loadings were excluded. This was also done so that information could be presented most concisely.

Using the codebook, each transcript was double coded individually, and discussions and iterative reviews were conducted with senior team members (MS, SS) to resolve discrepancies. Through interactive discussions and consensus meetings, the CFIR ‘Engaging’ construct was divided into two sub-constructs to capture engagement with different stakeholders (school administrators, teachers and students). Likewise, the CFIR ‘Relative advantage’ construct was divided into two sub-constructs to capture the differences in face-to-face and Zoom implementation. S.G., W.J.O., and B.W.Z.L. developed a summary table for the barriers and facilitators for the relevant constructs. To determine the key salient barriers and facilitators, the three team members rated each construct using Damschroder and Lowery’s criteria (2013) as a guide (Table 2). The ratings are indicative of the strength and valence of each construct, ranging from −2 to +2, with zero representing no indication of an effect or mixed effects (Damschroder and Lowery 2013). To ensure methodological rigour, multiple strategies were used, including team-based coding, triangulation of data sources, and consensus-building discussions. These steps were critical in enhancing the credibility and relevance of the findings. Additionally, the team engaged in ongoing reflective practices to critically examine how personal assumptions, experiences, and disciplinary orientations could influence the process. This included regular debriefings during data collection and analysis, and the use of collaborative coding to ensure that multiple viewpoints were considered. No metrics calculation was used for intercoder reliability as the team prioritized the co-construction of meaning over quantifying coder agreement. Therefore, the team focused on achieving conceptual clarity through iterative coding development, double-coding, and consensus meetings, aligning with a process-oriented approach.

**Table 2:** CFIR construct ratings for the implementation of the DYM programme.

	CFIR construct	Rating
1. Intervention characteristics	Relative advantage: experiential sessions	+2
	Relative advantage: zoom sessions	−2
	Adaptability	−2
	Complexity	−2
	Design quality and packaging	+1
2. Outer setting	Client needs and resources	+2
	Cosmopolitanism	+1
	External policy and incentives	0
3. Inner setting	Networks and communication	+1
	Culture	+1
	Tension for change	+1
	Compatibility	0
	Relative priority	−1
	Organizational incentives and rewards	−1
	Goals and feedback	−1
	Available resources	−2
	Access to knowledge and information	+1
4. Characteristics of individuals	Self-efficacy	0
	Other personal attributes	0
5. Process	Planning	+1
	Engaging school administrators	+2
	Engaging students and teachers	−2
	Executing	+2
	Reflecting and evaluating	+2

### Quantitative

#### Participants

Pre-post data from 841 Secondary One students across six sessions at three schools were collected. Secondary One students (aged 12–13) are typically in their first year of secondary school, which they enter after completing six years in Primary school. The first school had 574 respondents across four sessions: two held in October 2020 (299 students) and two in March 2021 (275 students). Two additional schools held one session each in October 2021 (118 and 149 students, respectively). All participating schools were government-run public schools, with student demographics and enrolment sizes representative of typical Singaporean secondary schools. Each student attended one session. Informed consent was obtained from parents before the session.

#### Data collection

Students completed a pre-programme questionnaire designed specifically for this intervention, with 21 items developed in consultation with content experts, youth mental health practitioners, and programme facilitators to ensure contextual relevance and face validity. These items were constructed in alignment with the programme’s objectives. Specifically, the items were designed to assess: (1) knowledge of the four targeted mental health issues (e.g. ‘List two signs and symptoms of an eating disorder’), (2) empathy and acceptance towards individuals experiencing mental health issues (e.g. ‘I am willing to take the step to better understand the struggles of persons with mental health issues’), (3) help-seeking attitudes (e.g. ‘If I were to show preliminary signs and symptoms of mental health issues, I will seek professional help’), and (4) willingness to seek support from appropriate sources, including friends, family, and professionals, for mental health concerns. Items were scored individually on a 5-point Likert Scale with 1 indicating ‘Strongly Disagree’ and 5 indicating ‘Strongly Agree’, except two categorically scored items: ‘Please list down two signs and symptoms of depression’ and ‘Please list down two signs and symptoms of eating disorders’ (0 = Not being able to list any sign/symptom correctly, 1 = Able to list one sign/symptom correctly, 2 = Able to list two signs/symptoms correctly). Participant responses for the two items were scored by programme staff using the diagnostic criteria for Major Depressive Disorder and Eating Disorder as a guide. These disorders were assessed using open-ended questions because of the assumed knowledge gaps in 2020, where part of the student population might have used terms inaccurately. The responses provided an understanding of the terms that students were using in association with these two conditions (e.g. lonely or sad for depression, and not eating, eating too much, or eating too little for eating disorders). Conversely, this was not perceived for conditions such as self-harm and anxiety. Reliability testing revealed a good Cronbach’s alpha score of 0.85 and 0.90 for the 21-item pre- and post-questionnaires, respectively, indicating good internal consistency.

#### Data analysis

The pre-post survey was consolidated in an Excel spreadsheet and prepared for analysis by the organization. As part of this process, the organization matched the pre-post data for each student using a unique identifier, removed identifiers, checked for data accuracy, and rectified any data entry errors and missing values.

Statistical analysis was conducted by the research team using the Statistical Package for Social Sciences (SPSS) Version 29 and STATA MP Version 18.0. Non-parametric analyses were conducted to test for pre-post significance, as preliminary analysis revealed that the data were not normally distributed. Most of the variables were analysed using the Wilcoxon signed-rank test, with Cohen’s D to determine the effect size. The exceptions to that were two variables that contained students’ scores for listing two signs and symptoms of depression and eating disorder. For each outcome, an unconditional multinomial mixed model (without covariates) was generated to examine the difference in outcome pre- and post-intervention. The model was generated using the xtmlogit command in STATA/MP Version 18.0.

Additionally, a difference-in-difference (DiD) model was utilized to analyse pre-post differences between students who participated in a modified Zoom programme due to the COVID-19 pandemic and students who participated in the face-to-face programme. The DiD model compares the changes in the survey results of students who participated in the Zoom programme, both before and after the programme, whilst accounting for any changes in the students who participated in the face-to-face programme. An unconditional linear mixed model with interaction terms (Delivery format × pre-post) was generated to determine the moderating effect of delivery format on the outcomes at pre-post intervention. An alpha value of 0.05 was used for all analyses.

## Results

### Qualitative data

The qualitative data were derived from 22 stakeholders across the three stakeholder groups (organisation staff, school liaisons and mentors) and six observation sessions. Table 3 reflects participating stakeholders’ sociodemographic information. Across the CFIR domains, seven constructs (two of which were divided into sub-constructs) were identified as most salient. Minor modifications were made to the construct names to enhance understanding in the context of this implementation. The most salient facilitators were the Relative advantage of VR experiential sessions, Student mental health needs and resources, Engaging school administrators, Executing with fidelity and contextual fit, and Reflecting and evaluating. The most salient barriers were the loss of the Relative advantage during Zoom sessions, Complexities during execution, insufficient Available resources, and Engaging students and teachers.

**Table 3:** Sociodemographic characteristics of participants.

	Mentors (*N* = 10)	School liaisons (*N* = 4)	Organization staff (*N* = 8)
Sex, *N*			
Female	10	4	5
Male	0	0	3
Age in years, Mean (range)	25.70 *(22–35)*	42.75 *(27–53)*	28.88 *(25–34)*
Ethnicity, *N*			
Chinese	9	3	8
Malay	0	1	0
Indian	1	0	0
Educational level			
Post-graduate degree	2	1	2
Bachelor’s degree	5	3	6
Polytechnic diploma	1	0	0
A level/completed Pre-U or Junior College	2	0	0
Educational background			
Mental health related^a^	5	2	6
Non mental health related	3	2	1
No of DYM sessions attended, mean (range)	8.90 (2–15)	—	—
No of stations involved in		—	—
1 station	2	—	—
2 stations	2	—	—
3 stations	4	—	—
All stations	2	—	—
No of months involved in DYM, mean (range)	—	—	19.63 *(7–36)*

^a^Mental health-related educational background includes social work, counselling, and psychology.

### Significant facilitators

#### Relative advantage of VR experiential sessions

School liaisons identified the experiential learning mode of the programme as advantageous over a typical school assembly lecture. The VR component, being relatively uncommon in similar programmes, helped capture schools’ attention and interest. Most school liaisons were unaware of comparable school-based mental health programmes, positioning the organization and its programme as the prime choice. The interactive facilitation design, compared to mass assembly talks, enabled staff and mentors to better engage with students and provided them with a more conducive space to discuss sensitive topics and approach mentors for any assistance.

‘I’m not aware, at least of any other community partners running a programme in such a style of hands-on experiential and VR. So, they would be the only ones really for this sort of engagement for students.’—[School Liaison Officer, SL03]

‘We also liked that there were a variety of activities which fit in with preference for something that was more hands-on for the students, as this would facilitate learning.’—[School Liaison Officer, SL03]

#### Student mental health needs and resources

The organization identified raising mental health awareness among youths and educating them on mental health resources and coping mechanisms as primary needs for this group. This was based on their observation that mental health issues amongst this population are on the rise, coupled with delayed treatment. The organization also noted that these students needed guidance on help-seeking avenues.

‘Mental health has always been an issue in society. I think clearly it has been gaining ground a bit. So, in developing the programme specifically, its mental health education, and we are coming in early with secondary school kids to educate them on what mental health actually is, to debunk some of the myths they had …’—[Organisation Staff, TS04]

Schools were similarly aware of the need to educate their students on such issues.

‘… I wanted youths to be aware of the mental health issues, what were the symptoms, who they can seek help from and importantly, knowing how to help … to know when to bring their friend to a teacher or school counsellor if they notice worrying signs and symptoms…’—[School Liaison Officer, SL02]

Schools also highlighted that the organisation’s staff considered factors that were helpful in engaging students and utilized these methods (e.g. use of VR) to pique their interest.

‘Our students are very energetic, so them moving around actually makes it interesting. Then every station has a different activity … using VR to introduce mental wellness to them, I think it might intrigue them and then make them more engaged.’—[School Liaison Officer, SL04]

#### Engaging school administrators

The organization used various means to engage schools, including via email blasts, events, and roadshow booths, which allowed them to raise awareness and market their programme. Furthermore, the booths allowed the organization to showcase their VR activity to school staff, enabling them to understand how this activity would contribute to their students’ mental well-being.

‘… like last year, we have a lot of road shows in different general public events. … Some of the schools will show interest, and then we will kind of connect from there… so I think some of the road shows, some of the mental health-related events, and some conferences… we will set up our booth there and showcase our technology and VR …’—[Organisation Staff, TS01]

#### Executing with fidelity and contextual fit

The programme was mostly executed as planned and was carried out well. The organization was able to identify core content for the four topics, and mentors maintained fidelity throughout the sessions. Although the core content was standardized, mentors had flexibility in how they delivered it, as long as the information remained accurate. They varied their delivery to suit the audience and group dynamics.

‘We try our best to uniform the content. So, we don’t actually take a lot of liberty through the content, we try to stick close to it. So, any adjustments that we make, minor and contextualised, maybe to the classroom, to the age group. Maybe we’ll switch up the use of examples that are more relevant to us, or maybe class-specific’—[Mentor, MT01]

Mentors also stated that delivering the content was made easier with the manual to follow as a guide.

‘Every mentor receives the slides, like the content to be delivered as well as the verbatim (guide) that shares what can be said … that was quite helpful’—[Mentor, MT10]

#### Reflecting and evaluating

The organization actively monitored and evaluated the programme, collecting feedback from students and teachers involved and sharing it internally. Debriefing sessions were conducted with mentors after each run, and the team continuously incorporated feedback to strengthen delivery. Staff and mentors were cognisant of the need to keep the programme responsive to youths’ evolving needs and to take steps to enhance it over time.

‘I know there are a lot of plans to improve and enhance it … They (The Impact & Research Team in the organisation) will try to churn out new ideas or information or stuff to include into the session to kind of like cater to all the enquiries.’—[Organisation staff, TS05]

### Significant barriers

#### Loss of relative advantage during zoom sessions

Due to COVID-19 safe management measures, face-to-face sessions could no longer be conducted. The organization modified the programme to be delivered via Zoom. However, the programme was unable to retain most of its interactive activities in the online setting, thus losing its relative advantage over other school-based mental health programmes.

‘I do not think it’s as effective as originally planned … If I were to judge it as a talk, with all other talks on mental health, I would say probably it performed not as well as others.’—[School Liaison Officer, SL03]

#### Complexities during execution

Logistical and technical (IT) issues were the biggest challenge to running the programme. Firstly, technical difficulties (e.g. VR equipment malfunctioning) and a lack of readily available technical support from schools caused significant delays.

‘Sometimes (the VR sets) get disconnected, and the other students get held back because we need to take a lot of time to reconnect again.’—[Mentor, MT04]

Secondly, logistical issues such as scheduling with the schools, time constraints, and limited infrastructure and space within schools also served as salient challenges.

‘Some of the difficulties were really, I would say, space constraints’—[School Liaison Officer, SL03]

‘Every time there is like some round robin station activities, so if we overrun, then the other station will be compromised also. The weakness itself honestly, I think is time.’—[Mentor, MT01]

#### Insufficient available resources

It was consistently mentioned by both mentors and staff that there was insufficient manpower for the implementation of the programme.

‘I think there’s always a struggle whereby the staff cannot find manpower or they cannot find volunteers.’—[Organisation Staff, TS7]

This led to issues such as not having additional personnel on hand to assist the mentors with their individual stations and challenges in pairing senior staff with new mentors for real-time mentoring. Manpower shortages stemmed from low volunteer sign-ups due to scheduling conflicts and pandemic restrictions, alongside a lean core programme team. The programme had to often tap on staff from other departments in the organization.

‘So, of course it will be helpful to always have another person but because we do rotation basis, and we try to keep like each station like small … Because too big of a group, people will be distracted, people will be talking and stuff … but smaller groups will also require us to have more manpower.’—[Mentor, MT01]

#### Challenges in engaging students and teachers

Observation data indicated that mentors and staff had difficulty engaging all students fully. Despite their best efforts, there were groups of students who were often not participative, distracted, and sometimes disruptive.

‘Not all students participated. Supposedly, students in blindfolds should be picking up the matchsticks, but some blindfolded students just remained stationary leaning against the wall.’—[Observation, Site 3]

Furthermore, classroom management was challenging. There was some indication that some teachers were not very involved when the organization’s staff were onsite, which hindered smooth delivery when their support was needed.

‘Teachers for this session played a particularly passive role … they did not manage the students when they had gotten fidgety or started walking around.’—[Observation, School 5]

### Quantitative assessments

Wilcoxon signed-rank tests indicated that pre- to post-program score differences were statistically significant across all measured variables. Students scored better in terms of their attitudes and knowledge of mental health issues after attending the programme. Effect sizes ranged from small (e.g. 0.19 for the item ‘Mental health issues can affect anyone like us’) to large (e.g. 0.95 for ‘I know how to help persons with mental health issues). The varying effect sizes reflect that some aspects of the programme were more beneficial in increasing MHL as compared to others. The breakdown of pre-post items is reflected in Table 4. The unconditional multinomial mixed model revealed that significantly more individuals reported two signs and symptoms of both depression and eating disorders post-intervention. Firstly, as compared to 0 signs and symptoms at pre-intervention, participants were more likely to report two signs and symptoms of depression at post-intervention (OR: 2.16, 95% CI: 1.51–3.07). Secondly, as compared to 0 signs and symptoms at pre-intervention, participants were more likely to report two signs and symptoms of eating disorder at post-intervention (OR: 1.54, 95% CI: 1.14–2.08). The frequency of participants who scored 0, 1 or 2 during pre-test and post-test for both variables is reflected in Table 5.

**Table 4:** Analysis of all Likert-scale variables pre- and post-programme.

Item	*N*	Mean (pre)	Mean (post)	Difference in mean	Cohen's d	*P*-value
1. Mental wellbeing is important to me	841	4.4	4.61	0.21	0.36	<.001
2. I am willing to interact with persons with mental health issues	841	4.23	4.46	0.23	0.29	<.001
3. I believe mental health issues can be treated	840	4.18	4.61	0.43	0.56	<.001
4. Mental health issues can affect anyone like us	841	4.62	4.74	0.12	0.19	<.001
5. Self-harm is a good way to cope with my emotions^a^	840	4.45	4.63	0.18	0.22	<.001
6. Anxiety is different from stress	839	4.11	4.48	0.37	0.39	<.001
7. I feel comfortable in interacting with persons with mental health issues	841	3.89	4.3	0.41	0.44	<.001
8. I know how to help persons with mental health issues	841	3.1	4.14	1.04	0.95	<.001
9. I am willing to take the step to better understand the struggles of persons with mental health issues	840	4.3	4.51	0.21	0.27	<.001
10. If I were to show preliminary signs and symptoms of mental health issues, I will seek professional help	839	3.55	4.02	0.47	0.47	<.001
11. Approach FAMILY for help if you are struggling with mental health issues	839	3.69	3.88	0.19	0.24	<.001
12. Approach FRIENDS for help if you are struggling with mental health issues	840	3.83	4.03	0.2	0.26	<.001
13. Approach SCHOOL COUNSELLORS for help if you are struggling with mental health issues	840	2.65	3.15	0.5	0.49	<.001
14. Approach TEACHERS for help if you are struggling with mental health issues	840	2.61	2.96	0.35	0.36	<.001
15. Approach PRIVATE COUNSELLORS for help if you are struggling with mental health issues	840	2.86	3.26	0.4	0.39	<.001
16. Approach DOCTOR for help if you are struggling with mental health issues	840	3.02	3.23	0.21	0.21	<.001
17. Approach PSYCHIATRIST for help if you are struggling with mental health issues	839	3.08	3.46	0.38	0.39	<.001
18. Approach HOTLINE for help if you are struggling with mental health issues	840	2.33	2.93	0.6	0.53	<.001
19. Approach SOCIAL MEDIA/ONLINE FORUM for help if you are struggling with mental health issues	840	2.16	2.39	0.23	0.23	<.001

*Note*. ^a^1 = Strongly agree, 5 = Strongly disagree.

**Table 5:** Frequency of scores of 0, 1, or 2 during pre-intervention and post-intervention for the two remaining variables.

	Frequency (%)	
Please list down two signs and symptoms of depression	Pre	Post
Score of 0	134 (16.5%)	132 (15.7%)
Score of 1	349 (42.9%)	254 (30.2%)
Score of 2	331 (40.7%)	455 (54.1%)
Please list down two signs and symptoms of eating disorder	Pre	Post
Score of 0	190 (22.6%)	158 (18.8%)
Score of 1	284 (33.8%)	273 (32.5%)
Score of 2	367 (43.6%)	410 (48.8%)

#### Differences in outcomes between methods of delivery

**Figure 1:**
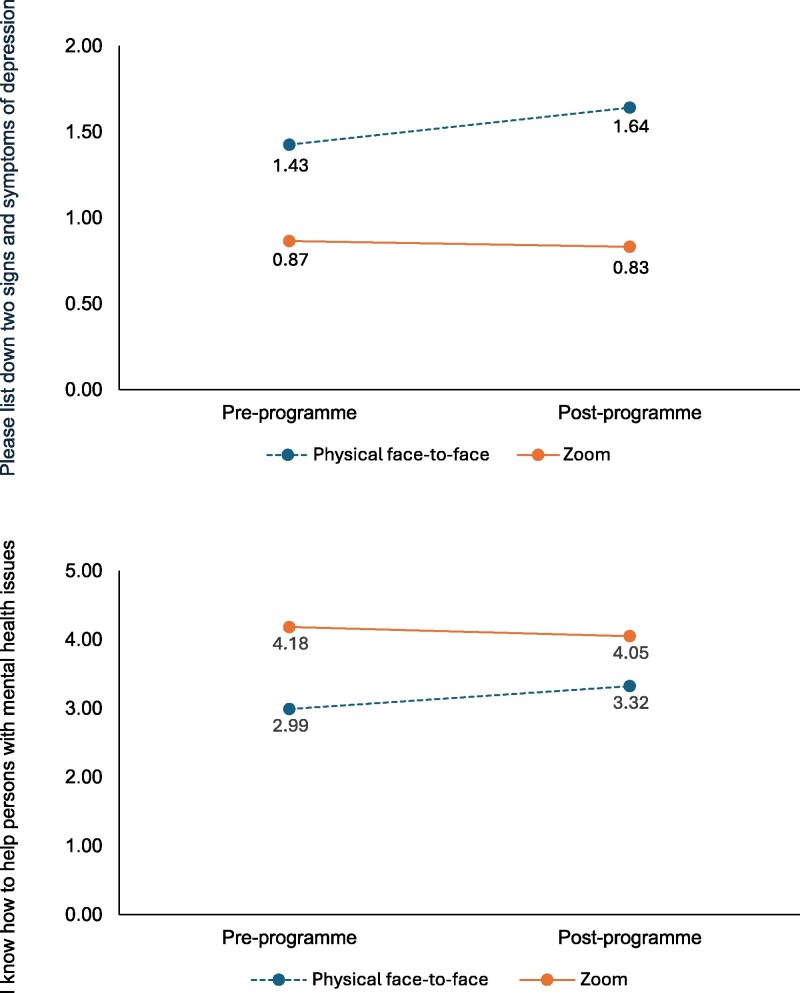
Difference-in-difference graphs depicting the differences in pre-programme and post-programme scores for the variables ‘Please list down two signs and symptoms of depression’ and ‘I know how to help persons with mental health issues.’

In the DiD analysis, face-to-face sessions showed significantly greater improvements than Zoom sessions for two items after controlling for pre-intervention outcomes. Specifically, face-to-face sessions had a 0.25 increase on the outcome ‘List two signs and symptoms of depression’, and a 0.47 increase on the outcome ‘I know how to help persons with mental health issues’ than Zoom sessions after controlling for pre-intervention outcomes. The results are depicted in Fig. 1. There were no significant differences between the two delivery modes for other items.

## Discussion

The present study aimed to evaluate the real-world implementation of a novel school-based MHL programme and explore its perceived impact. This was done through examining barriers and facilitators to implementation using a structured implementation framework, alongside assessing pre-post changes in students’ MHL for the program’s pilot run. The qualitative results provided insights into the implementation process, identifying key factors that influenced its delivery and uptake. One key facilitator was the programme’s relative advantage, which was central to its inception. VR has increasingly been used in education over the past decade and has been shown to enhance knowledge, empathy, and attitudes towards mental illness (Wan and Lam 2019, Aiello *et al*. 2012, Asad *et al*. 2021, Fromm *et al*. 2021, Hamilton *et al*. 2021). This element stood out to school liaisons and strengthened the programme’s appeal.

Additionally, understanding student mental health needs and resources was another key facilitator driving the programme’s inception. Both the organization and schools involved were cognisant of their target audience and the need to address mental health through a medium that optimized learning. Studies emphasize the need for messages to be tailored to the audience, as adolescents’ needs may differ from the general population (Seedaket *et al*. 2020 ). The organization, therefore, focused on core content addressing mental illnesses most relevant to adolescents, while considering their current MHL to design effective literacy programmes (Mendenhall *et al*. 2014, Seedaket *et al*. 2020). The organization also considered local data on youth MHL when designing the programme, ensuring that common knowledge gaps were addressed (e.g. Pang *et al*. 2017). In addition, schools involved were aware of the mental health needs of students, which made it easier to secure support. In a systematic review assessing barriers and facilitators for school-based implementation, school staff’s recognition of an intervention’s value to students was identified as a key facilitator for successful implementation (March *et al*. 2022). Continuing regular monitoring and reviewing of student MHL needs can help the organization to identify shifts over time and adapt and update programme design and content to meet new needs.

Furthermore, the organization engaged schools through indirect and direct outreach efforts, using various platforms such as emails and roadshows. Multiple studies have highlighted that school support is key to implementation success, making it crucial to engage stakeholders like administrators and secure their buy-in (Domitrovich *et al*. 2008, Pronovost *et al*. 2008, Damschroder *et al*. 2009, Langley *et al*. 2010, Richter *et al*. 2022). Additionally, engaging stakeholders throughout implementation helps to improve communication, especially when decision-making is involved (Richter *et al*. 2022).

During implementation, feedback from mentors, staff, and observation data consistently indicated that the programme was executed as intended, with the manual guides being helpful in delivering content. Uniform delivery can be challenging in schools due to varying student profiles and classroom environments (Durlak and DuPre 2008, Combs *et al*. 2022). Effective implementation requires balancing fit and fidelity (Anderson 2017, Wiltsey Stirman *et al*. 2019). Mentors modified content to suit student needs and classroom dynamics, while adhering to core components. For successful delivery, facilitators must recognize which elements cannot be adhered to and be able to adjust the rest to suit contextual factors such as student demographics (McHugo *et al*. 2007, Harn *et al*. 2013). Organizations that adapt their programme to match these contexts are more likely to sustain their programme in the long run (Swain *et al*. 2010). There is an increasing emphasis that modifications based on student needs allow for better intervention outcomes (Webster-Stratton *et al*. 2011, Harn *et al*. 2013).

However, for some schools, there were barriers engaging students and teachers during programme implementation. Disruptive student behaviours, poor classroom management, and limited teacher support have been reported to undermine successful programme delivery (Langley *et al*. 2010, Koester *et al*. 2021). Mentors not trained in classroom management reported low efficacy in managing disruptive student behaviour. Langley *et al*. (2010) similarly ranked lack of support from teachers among the top implementation barriers of their mental health programme. Effectively engaging teachers supports delivery as teachers and students often have pre-existing rapport (Baweja *et al*. 2016, Chodkiewicz and Boyle 2017). In a sense, teachers serve as external agents whose support can steer the programme in a desirable direction. Classroom management strategies, like using posters with cue words to set behaviour expectations, have been shown to help students transition smoothly between activities (Beemer *et al*. 2018). Using such strategies may help promote student cooperation for future implementations.

Another salient barrier to the implementation pertained to logistical complexity. Implementation of external programmes in schools is challenging due to scheduling sessions within the constraints of timetables and availability of spaces (Langley *et al*. 2010, Hudson *et al*. 2020, Gee *et al*. 2021). Implementation was affected by limited support for school audio-visual equipment and third-party VR systems. Such issues echo findings from Kaimara *et al.* (2021) that identified outdated technological equipment as a major barrier to implementing digital learning in Greek classrooms. Similarly, a scoping review of 30 studies on digital mental health interventions for children and adolescents reported that technical issues commonly hindered their use (Bergin *et al*. 2020). However, these issues were overcome when adequate support was provided to those delivering the intervention (Bergin *et al*. 2020, Kaimara *et al*. 2021). As such, for future implementations, it is important to obtain sufficient on-ground support from schools.

Additionally, insufficient manpower to support delivery was found to affect various areas of implementation. For some mentors, the programme was not part of their primary job responsibilities and frequent enlistment to help in the programme increased their workload. Hartmann *et al*. (2021) suggested that assigning clear facilitation roles is the key to ensuring smooth programme delivery. However, this was not possible considering the limited manpower. Moreover, manpower shortage resulted in less experienced staff members delivering sessions independently. This challenge was also noted in other school-based interventions as a major barrier to effective delivery and sustainability (e.g. Forman *et al*. 2009). Forman *et al*. (2009) cautioned that dependence on a small facilitator pool is unsustainable, as they may not remain available to support the programme long-term.

In short, impediments such as manpower shortage, technical difficulties, and classroom management challenges were reported during the implementation. Given that the participating schools had a typical profile of local secondary schools, these highlight general implementation barriers, underscoring the importance of addressing such factors to optimize programme delivery. Proactively mitigating these challenges might also enhance the scalability and sustainability of the programme across a broader range of educational settings.

Lastly, to understand the factors that were challenging or helpful, the organization utilized an incremental approach with constant revisiting, refining, and evaluating (Damschroder *et al*. 2009). Traditional pre-post feedback and outcome data were collected from students to assess delivery and effectiveness. Debriefs were conducted by staff with mentors and school liaisons post-sessions to capture a wider range of perspectives. Mentors and staff continuously shared feedback throughout implementation, addressing obstacles to refine delivery. This constant reflecting and evaluating serves as a critical component for the sustainability of the programme.

Extending these qualitative insights, the quantitative results revealed that participants improved in all assessed outcomes from pre- to post-intervention, indicating the potential of the programme in enhancing MHL. These improvements align with existing evidence that targeted interventions can effectively strengthen MHL among younger adolescent populations (Zachik *et al*. 2025). It is also worthwhile to note that the pattern of effect sizes for pre-post scores appears to be influenced by participants’ baseline levels of MHL. Overall, items with lower baseline scores exhibited larger pre-post gains, while items with higher pre-scores displayed a smaller effect size. This trend suggests that larger shifts occurred in areas where participants started with lower knowledge. Future iterations could use baseline MHL levels to refine content and devote more time and resources to topics where prior knowledge is weakest.

Both delivery modes resulted in similar changes in participants’ MHL, with no significant difference in most items, indicating that Zoom can be just as effective as in-person delivery for most components. However, two items showed notable differences in favour of face-to-face delivery. Qualitative feedback helped to contextualize this finding, as stakeholders reported that the in-person session fostered greater engagement and richer interactions. A key factor underlying this discrepancy is the absence of the VR interactive component in the online sessions. The VR module, a core part of the programme, provides an immersive, first-person experience of mental health challenges in simulated scenarios. Participants scored higher on the item ‘Please list down two signs and symptoms of depression’ in the in-person sessions, likely due to the VR simulation on depression, which was not offered for other mental health conditions in this pilot run. By removing this interactive feature in the online format, participants likely had fewer opportunities for experiential engagement, which might explain the lower scores.

Overall, these findings suggest that face-to-face delivery, enhanced by VR, provides a distinct advantage. Nevertheless, the comparable outcomes in most items highlight the viability of online delivery for broader scalability and accessibility, particularly in contexts where in-person sessions are not feasible. Future iterations of the programme can explore hybrid models to bridge the gap between both formats.

## Limitations and strengths

Several limitations of this study should be considered. Firstly, findings were based on participants who agreed to participate in the research, introducing potential self-selection bias, as their experience may differ from that of non-participants. Secondly, participants may have viewed the study as an evaluation of their performance, potentially giving socially desirable responses, despite assurances otherwise. Similarly, the presence of observers, though minimally disruptive, could have induced a Hawthorne effect, altering participants’ behaviour (Cook 1962). Another limitation is the absence of adjustment for multiple comparisons. Given the ongoing debate about the necessity of corrections such as Bonferroni, which are often criticized for being overly conservative and inflating Type II errors, we chose to retain the conventional significance level (*P* < .05) (O’Keefe 2003, Greenland and Hofman 2019, Rubin 2021). Nonetheless, even with conservative adjustments, the findings would remain statistically significant as all main outcomes were highly significant (*P* < .001). Additionally, effect sizes (Cohen’s D) were considered in the interpretation of the results to help convey their practical significance (Cumming 2014, Wasserstein and Lazar 2016), providing a balanced interpretation beyond *P*-values.

Additionally, the absence of a control group should also be noted. A non-treatment comparison was not feasible due to ethical concerns about withholding MHL content and practical constraints within schools’ implementation schedules. Additionally, ethical regulations did not allow data collection from non-participating schools. Consequently, the study relied on a pre-post, within-subject design, which limits causal inference. This study design was utilized because the primary aim was to assess how the programme was delivered in real-world settings and to explore its perceived impact, rather than to determine causal effectiveness. Given the positive changes observed in MHL, future research could strengthen these interpretations by using a stepped-wedge or cluster-controlled design, for example, once feasibility, scaling and ethical considerations have been addressed.

Notwithstanding its limitations, a key strength of the study lies in its mixed-methods design and the use of a systematic coding approach. Mixed methods research can enhance the rigour of implementation research by integrating empirical data on the effectiveness of interventions with data that explains the reasons behind efficacy. Additionally, a systematic qualitative coding approach ensured analytical rigour and transparency through team-based coding, triangulation of data sources, and consensus-building discussions. This method allowed us to capture the contextual nuances and mechanisms that influence the implementation process. This approach enhances the validity and comparability of findings across studies, while also generating data that can possibly inform practice.

## Conclusion

The findings suggest important areas to focus on to maximize the implementation of school-based mental health programmes. Quantitative student outcomes supported qualitative findings on intervention barriers and facilitators, suggesting that the hands-on, immersive approach enhanced student learning. Building on this strength, future iterations of the programme could expand VR use to additional mental health topics to further enhance learning. Despite certain barriers, student outcomes were positive post-programme, suggesting inherent strengths that supported the programme’s potential effectiveness. It is important that implementation research findings like these are disseminated in forms that are accessible and appropriate to service providers (Challen *et al*. 2014), so that challenges and facilitators can be anticipated and addressed to improve future implementation.

## Supplementary Material

daaf236_Supplementary_Data

## Data Availability

Individual transcripts, observation notes, and the dataset are not available to the public due to confidentiality policies.
