# Supplementary material for: Implementing a school-based mental health literacy programme for adolescents: barriers, facilitators and preliminary outcomes
Source: Health Promot Int. 2026 Jan 13;41(1):daaf236. doi: 10.1093/heapro/daaf236 (PMC12798539; doi:10.1093/heapro/daaf236)
Supplement: daaf236_Supplementary_Data [file daaf236_supplementary_data.zip › Do You Mind Supplementary Material 1.docx]

**Brief Intervention Outline**

| **Duration** | **Sequence of Activities** |
| --- | --- |
| 15min | **Introduction to the programme and the importance of mental health**  The session’s overall mentor in charge (staff) shares contextual information on the prevalence of youth mental health issues, followed by the key learning objectives and ground rules. That includes encouraging students’ participation in station activities to learn more about mental health while respecting the confidentiality of the information shared by their peers.  Students are assigned into smaller groups of between 15-20 pax via the use of the coloured wristlets distributed by the overall facilitator and mentors. This is intended to encourage students’ attention and learning in a more conducive setting as compared to a large group. |
| 150min | **Commencement of station activities**  (Number of stations = 5)  Each group takes turn to participate in the five stations (Depression, Anxiety, Eating Disorders, Self-Harm, and Virtual Reality Experience on Depression).  Each station will take around 25-30 mins, including briefing and debriefing. There will be 1-2 mentor(s) who facilitate the station activity, guided by the resource package with verbatim provided to ensure consistency and quality in their delivery. Each station features experiential activities aligned with the mental health condition introduced^1^.  Moving from station to station will take around 3 minutes.  Pre-survey forms will be completed by the students and collected by mentors at their first station before the station activity starts.  Post-survey forms will be completed by the students at their last station upon the completion of the station activity. The forms will be collected by the mentors to facilitate subsequent data entry. |
| 15min | **Ending segment of the programme**  The mentor in charge (staff) provides a summary to recap the session’s key mental health content and encourages students to reflect on their learning experience and how they intend to apply what they have learned.  Mental health services and resources, including complimentary counselling services provided by the organisation and the participating school (e.g. school counselling services), are reiterated to normalise and encourage health-seeking behaviours, particularly among students who need more support for their mental health.  The mentor-in-charge conducts Q&A to address any other concerns and feedback that the students may have. |
| Total Duration: 180-210 mins | |

^1^Experiental activities were not conducted during Zoom sessions except for the Anxiety station due to feasibility constraints in a non-in-person setting.
