# Supplementary material for: Implementing a school-based mental health literacy programme for adolescents: barriers, facilitators and preliminary outcomes
Source: Health Promot Int. 2026 Jan 13;41(1):daaf236. doi: 10.1093/heapro/daaf236 (PMC12798539; doi:10.1093/heapro/daaf236)
Supplement: daaf236_Supplementary_Data [file daaf236_supplementary_data.zip › Do You Mind Supplementary Material 2.docx]

**Examples of interview questions for each stakeholder group and observation guide questions**

| **Stakeholder Group** | **Questions** |
| --- | --- |
| Mentors | - Do you feel supported enough by the organization in carrying out your roles? And, are there any other things you could think of that the organization could provide you with to enhance your performance? [Inner Setting: Access to knowledge & Information] - Are there any sorts of incentives in place, such as recognitions or awards, perhaps to motivate you in your work? Besides organizational incentives, do you have any personal motivation to see the DYM work, and why? [Inner Setting: Organizational Incentive & Rewards] - Overall, how prepared or confident do you feel in the role that you play in executing the DYM? [Characteristics of Individual: Self-efficacy] |
| School Liaisons | - How does DYM compare to other similar existing programs in your school, if any? What are DYM’s relative strengths or drawbacks? [Intervention Characteristics: Relative Advantage, Implementation Climate: Tension for Change] - There must be many activities going on in your school right now. How are you juggling your role as coordinator of DYM alongside your other work duties? What about your colleagues and school leaders – to what extent is the implementation of DYM a priority to them? [Inner Setting: Implementation Climate – Relative Priority] - Can you share with me the plans that you or your school has drafted regarding the implementation of DYM? How feasible do you think these plans are? [Process: Planning] - Have you gathered any feedback from students so far about their experience with the program? What do they like about the program, or what do they think can be improved? [Outer Setting: Patient Needs & Resources] |
| Organization Staff | - What kind of training was planned for you in preparation for this program? [Access to information and knowledge] - Since the initial development of the program, what changes in format or content did you have to make and why? [Adaptability] - How different or complex is the DYM compared to other programs you have been involved in? [Compatibility] |
| **School Observations** | **Point to Take Note** |
|  | - Did the mentor conform to the script verbatim? - Is the mentor clear, coherent and confident with the station verbatim guide? - Did everyone have access to the VR headset? - Did the mentor provide a complete and coherent summary of the station? - Is the mentor engaging and inciting interest? - Did the mentor encourage questions? - How many questions were asked by the audience? - Is the mentor clear, coherent and confident with their responses? - Did the mentor provide information on help-seeking avenues? - Describe any challenges that the mentors/staff faced. |

*Note.* CFIR constructs related to the interview questions are indicated in the brackets [ ].
